# Improving Catalytic Efficiency and Changing Substrate Spectrum for Asymmetric Biocatalytic Reductive Amination

**DOI:** 10.4014/jmb.1907.07015

**Published:** 2019-09-18

**Authors:** Wei Jiang, Yali Wang

**Affiliations:** 1 Department of Bioengineering and Biotechnology, College of Chemical Engineering, Huaqiao University, Xiamen, Fujian, 3602, P.R. China; 2 Department of Biochemistry, University of Washington, Seattle, WA 98195, USA

**Keywords:** Chiral compounds, asymmetric catalysis, substrate spectrum, biocatalysis, rational design, substrate binding pocket

## Abstract

With the advantages of biocatalytic method, enzymes have been excavated for the synthesis of chiral amino acids by the reductive amination of ketones, offering a promising way of producing pharmaceutical intermediates. In this work, a robust phenylalanine dehydrogenase (PheDH) with wide substrate spectrum and high catalytic efficiency was constructed through rational design and active-site-targeted, site-specific mutagenesis by using the parent enzyme from *Bacillus halodurans*. Active sites with bonding substrate and amino acid residues surrounding the substrate binding pocket, 49L-50G-51G, 74M,77K, 122G-123T-124D-125M, 275N, 305L and 308V of the PheDH, were identified. Noticeably, the new mutant PheDH (E113D-N276L) showed approximately 6.06-fold increment of *k_cat_*/*Km* in the oxidative deamination and more than 1.58-fold in the reductive amination compared to that of the wide type. Meanwhile, the PheDHs exhibit high capacity of accepting benzylic and aliphatic ketone substrates. The broad specificity, high catalytic efficiency and selectivity, along with excellent thermal stability, render these broad-spectrum enzymes ideal targets for further development with potential diagnostic reagent and pharmaceutical compounds applications.

## Introduction 

As vital pharmaceutical ingredients, biocatalysts are increasingly vogue in the large-scale biosynthesis of enantiomerically pure compounds (EPCs) [1-7]. Pure chiral compounds such as l-*tert*-leucine (l-Tle) and amine compounds are sought-after building blocks in pharma and are mainly used as active pharmaceutical ingredients (APIs) applied in such drugs as Reyataz (HIV), Telaprevir (HCV), oseltamivir (Tamiflu), rasagiline (Azilect) and sitagliptin (Januvia) [3, 8, 9]. Furthermore, transaminase catalysis is an accessible method that has recently been applied in the synthesis of sitagliptin [10]. The use of APIs increased by 35% from 2000 to 2010 [11], however, the appropriate biocatalysts were required for the reactions. The Pharmaceutical Roundtable noted that the asymmetric synthesis of chiral compounds, especially chiral amine compounds, from free NH3 and prochiral ketones, was one of the top aspirational routes challenging the pharmaceuticals industry, according to one recent assessment by the ACS Green Chemistry Institute [12]. Amine Dehydrogenase (AmDH) is the key enzyme in enzymatic production of amines [13-19], and suggests the significance of obtaining an enzyme with the properties of catalytic synthetic amine compounds by effecting reductive amination of ketones.

As the best candidate for providing a skeleton, the amino acid dehydrogenase scaffold, especially phenylalanine dehydrogenase (PheDH) and leucine dehydrogenase (LeuDH), is widely used for the development or creation of novel enzymes with the properties of catalytic synthetic amine compounds, such as AmDH [1, 2, 10, 11, 20]. Phenylalanine dehydrogenase (E.C. 1.4.1.20) catalyzes the reversible NADH-linked and reductive amination of phenylpyruvate to L-phenylalantne [21]. PheDH has been received much attention since it was discovered, as it is a greatly superior biocatalyst in the biosynthesis of phenylalanine, which is related to l-amino acids in the food industry and pharmaceutical peptides in the pharmaceutical industry [22-24]. PheDH has also been extensively applied in diagnostic kits and biosensors to detect blood serum of neonates [25] for phenylketonuria (PKU). However, the wide medical and biotechnological applications of this enzyme are frequently hampered by its limited enzyme activity, and the development of a PheDH with high activity based on natural enzymes is an efficient approach to improve the catalytic properties.

Although some chiral compounds have been produced en masse through asymmetric reductive amination of ketones by organometallic catalysts, the protocols that can lead to high enantiomeric excesses with the advantages of simplicity, low cost and environmental friendliness still require further exploration [20]. Hitherto, few studies have dealt with asymmetric synthesis of amines by starting with prochiral ketones [1,2,20,^2^6-28], probably due to the low activity and poor stability of the new-found AmDH and the complicated process. Accurate active-site residues have never been affirmed using kinetic analyses of the mutant and its parent. Structure-based, site-directed mutagenesis is usually applied to generate variants with immensely improved specificity and broad substrate spectrum; the residues in or near the active center tend to be chosen for their specific roles in the activity of the enzymes [29-31].

Herein, by using a PheDH scaffold of the marine microorganism *Bacillus halodurans*, we have successfully improved its catalytic efficiency and altered the substrate specificity to create a novel dehydrogenase through rational design and site-directed mutagenesis. The amino acid residues surrounding the substrate binding pocket were identified. Instead of the natural substrate, mutants now accept an analogous ketone, such as phenoxy-2-propanone, which catalyzes the reductive amination reaction, while the original PheDH exhibited no measurable activity for the reductive amination of other ketone compounds. Asymmetric synthesis by this new PheDH would be an ideal route to produce chiral compounds and natural products.

## Materials and Methods

### General

Chemicals were obtained from Sigma (Shanghai, China) and used without further purification. T4 DNA ligase, pMD18-T vectors, Taq DNA polymerase, restriction enzymes, DNA purification kit and gel extraction kit were all obtained from TaKaRa Co. (China). All oligonucleotide primers (Table S1) and fragments were synthesized and sequenced by Sangon Biotech (China). The marine bacterium *B. halodurans* (No. MCCC 1B00241) was provided from the Marine Culture Collection of China (MCCC) and cultivated at 30°C in 2216 medium. The plasmid, pET-28a-*pdh* containing the PheDH gene and sharing 100% identity with the *pdh* gene (1,140 bp, Gene ID: 893554) from *B. halodurans* C-125, was assembled.

### Site-Directed Mutagenesis

**Fig. 1:**
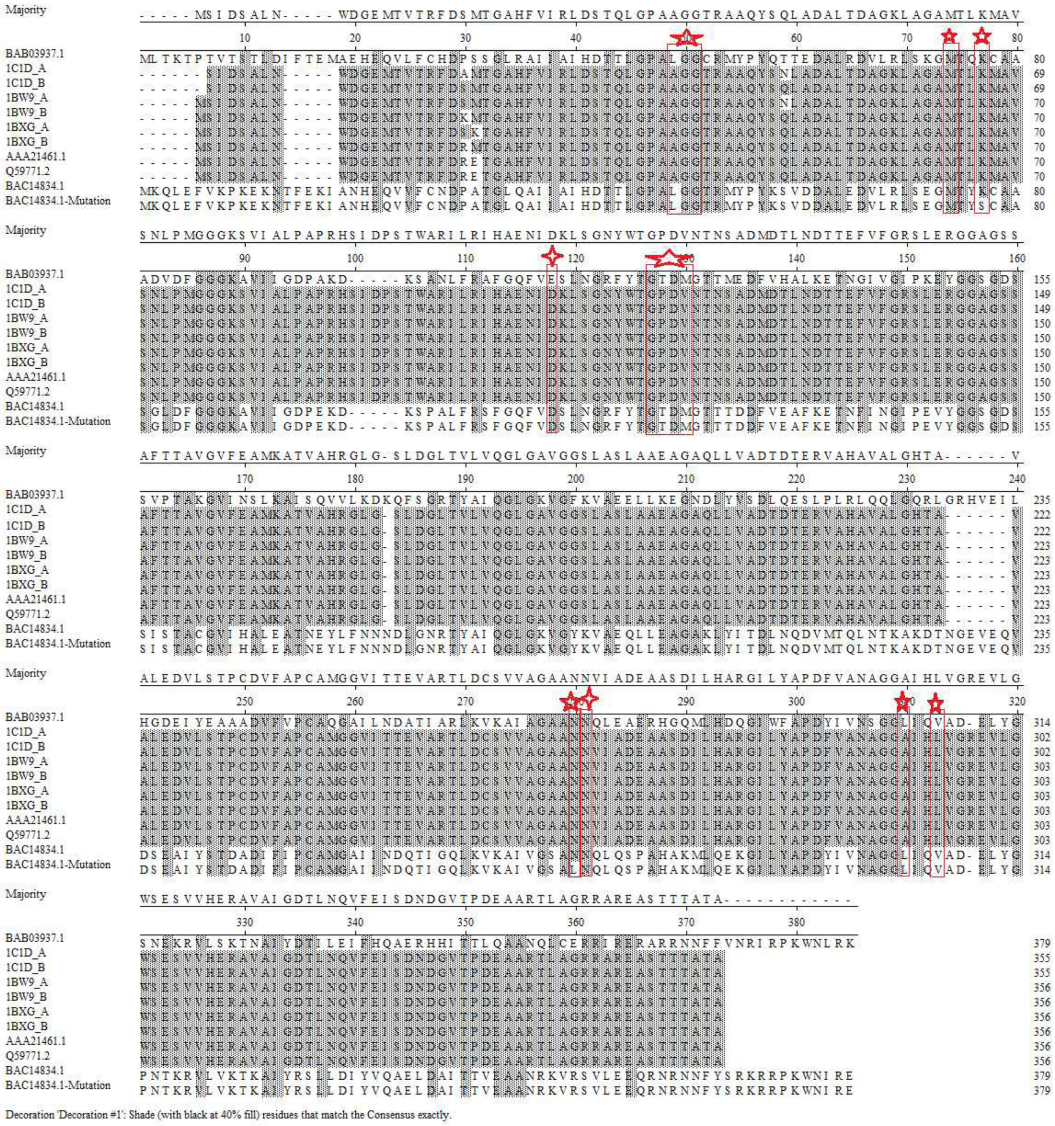
Sequence analyses of catalysis and substrate binding sites of the PheDH and its 10 most closely related structures in the PheDH family. The residues essential for PheDH catalysis and substrate binding are highlighted in red boxes and marked with five-pointed stars (red). The mutation sites are highlighted in red boxes and marked with quadrilaterals (red). Sequences, PheDHs from *Rhodococcus* sp. M4 or other species (Accession No. 1C1D_A, 1C1D_B, 1BW9_A, 1BW9_B, 1BXG_A, 1BXG_B, AAA21461.1 and Q59771.2) and PheDH from *Oceanobacillus iheyensis* (Accession No. BAC14834.1), were obtained from NCBI database (http://www.ncbi.nlm.nih.gov/guide/) and pre-reported (Brunhuber *et al*. 2000; Vanhooke *et al*. 1999).

**Fig. 2:**
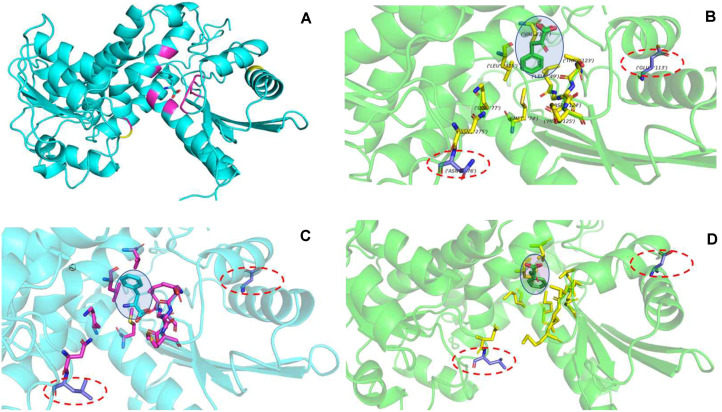
Binding mode analyses and structural models of the PheDH with phenylpyruvic acid or phenylalanine. (**A**) Structure analyses of catalysis and substrate binding sites (magenta) of the PheDH. (**B**) Binding mode analyses and structural models of the catalysis and substrate binding sites of the PheDH. The catalysis and substrate binding sites of (49L-50G-51G, 74M,77K, 122G-123T-124D-125M, 275N, 305L, 308V, and 89K) are shown in yellow. Introduced mutations (E113D and N276L) are shown in purple. (**C**) Docking of phenylalanine (shadow, cyan) into the binding pockets of the PheDH. The catalysis and substrate binding sites (49L-50G-51G, 74M,77K, 122G-123T-124D-125M, 275N, 305L, 308V, and 89K) are shown in magenta. Introduced mutations (E113D and N276L) are shown in purple. (**D**) Docking of phenylpyruvic acid (shadow, cyan) into the binding pockets of the PheDH. The catalysis and substrate binding sites (49L-50G-51G, 74M,77K, 122G-123T-124D- 125M, 275N, 305L, 308V, and 89K) are shown in yellow. Introduced mutations (E113D and N276L) are shown in purple. The three-dimensional structure was generated using the EasyModeller4.0. Phenylpyruvic acid and phenylalanine was docked into the structure of PheDH using AutoDock4.0 (http://autodock.scripps.edu) and Rosetta. The modeled substrate-enzyme complex was analyzed. The figure was generated using the program PyMOL.

The PheDH catalysis and substrate binding sites were confirmed to help obtain a mutant with superior performance and high activity. As the active sites and crystal structure of the *Rs*PheDH from *Rhodococcus* sp. M4 (PDB: 1C1D and 1BW9) have been reported [32, 33], it was selected as the template for the identification of the catalysis and substrate binding sites. This work aims to change the specificity of the substrate and enhance the catalytic efﬁciency of PheDH from *B. halodurans*. The secondary structure alignment of the *Rs*PheDH and PheDH was implemented, and a comparison of results is shown in Fig. 1. The corresponding sites in the *Rs*PheDH and PheDH active site with bound L-Phe and suitable surrounding residues were highlighted (Fig. 1). The alignments of the amino acid sequences of *Rs*PheDHs, the PheDH and other PheDHs were performed by ClustalW. In addition, the active site with bound L-Phe and suitable surrounding residues of the PheDH was also highlighted to show its three-dimensional structure (Fig. 2). According to the above results, two distinct sites (Figs. 1, 2, and S1) with Glu at position 113 and Asn at 276 were observed for the next experiment. Mutations are mostly concentrated on or close to enzyme activity sites, resulting in potentially more enhanced enantioselectivity or activity [7, 34]. According to the reported results, change on 113 and 276 amino acid residues would be most influential in the substrate specificity of the LeuDH-based scaffold from *Bacillus stearothermophilus* and the LeuDH-based scaffold from *Rhodococcus* sp. M4 [1, 35], while LeuDH and PheDH have high similarity.

Using the plasmid pET-28a-*pdh* containing the PheDH gene as the template, the site-directed mutagenesis was implemented by PCR as previously described [36] with moderate modifications. The primers containing the desired mutant are shown in Table S1 (Table S1). The positive plasmids were checked by sequencing, and the successfully introduced wishful mutation was designated as pET-28a-E113D and pET-28a-N276L. Then, the superposition of mutation pET-28a-E113D-N276L was obtained using the same method with the pET-28a-E113D or pET-28a-N276L as the template.

### Expression and Purification of Enzymes

The recombinant plasmids pET-28a-*pdh*, pET-28a-E113D, pET-28a-N276L, and pET-28a-E113D-N276L were transformed into BL21 (DE3) for the PheDH expression. Protein expression was induced by adding IPTG to a final concentration of 0.2 mM and the mixture was incubated at 22°C for 6 h. After that, the cells were harvested and disrupted. Then, the PheDHs were purified by the AKTA Prime system equipped with a 15-ml HisTrapTMFF column (GE Healthcare, USA). Finally, the purity of the enzyme was detected in 12% SDS-PAGE and the protein concentration was quantified using a Bradford Protein Assay Kit.

### Activity Measurements

Activity of purified enzymes was measured by a spectrophotometric assay at 340 nm, corresponding to the consumption or formation of cofactor NADH (ε=6,220 M^-1^cm^-1^) [37]. For reductive amination, reactions were implemented in 500 mM ammonia, 100mM glycine-KCl-KOH buffer (pH 10.4), 100 microM NADH, 20 mM of the ketone substrate and enzyme, unless otherwise specified. 1,4-dimethylpentylamine (CAS No. 28292-43-5) was R type. The enzyme activity for oxidative deamination was performed in 50 mM glycine-KCl-KOH buffer (pH 10.4) containing 2 mM NAD^+^ with 20 mM of the amine substrate of interest with moderate enzyme. All reactions implemented were performed at 25°C unless otherwise specified.

One unit (U) of enzyme activity was defined as the quantity of enzyme catalyzing the consuming or formation of 1 μmol NADH per min in the reductive amination of ketone substrate or oxidative deamination of the amine substrate, respectively, under the standard assay conditions. The variants were expressed for his-tag purification and measured in *k_cat_* and *K*_m_ values with phenylpyruvate acid, L-phenylalanine (like parent enzyme, the mutants had no activity on D-phenylalanine).

### Stability of Temperature and pH Value

The thermal stability of the enzyme was measured under the optimal pH (7.0 data no show) by pre-incubating the PheDHs at temperatures from 35°C to 70°C for 100 min and the residual enzyme activity was measured under the standard. The pH stability of the PheDHs was determined while it was incubated at 4°C for 48 h in different buffer systems (pH 4-9.5), and then the remaining activity was measured as described above. The biochemical characterization of the PheDHs was carried out with the same methods, except the buffers were 0.2 M acetic acid-sodium acetate buffer (pH 4-6.0), barbital sodium-hydrochloric acid buffer (6-9.0) and 0.05 M glycine-sodium hydroxide buffer (8.6-9.5) of the oxidative deamination with L-phenylalanine as substrate), and namely 0.2 M acetic acid, sodium acetate buffer (containing 200mM NH_4_Cl, pH 4-6.0) and 0.2 M NH_3_·H_2_O-NH_4_Cl buffer (6-9.5) of the reductive amination with phenylpyruvate acid as substrate.

### Computer Model Generation and Docking

The site of site-directed mutagenesis was indicated on the three-dimensional structure of PheDH, which was built from the known x-ray structure of the 1leh.1.A and 3vpx.1.A by manual modeling in EasyModeller4.0 [38]. Furthermore, the mutation sites were marked on the three-dimensional structure. Phenylpyruvic acid and phelalanine were docked into the structure of PheDH using AutoDock4.0 (http://autodock.scripps.edu) and Rosetta. The modeled substrate-enzyme complex was analyzed. The figure was generated using the program PyMOL.

## Results and Discussion

### Identification of Catalysis and Substrate Binding Sites

Amino acid dehydrogenases are known to share significant sequence similarities in the coenzyme binding catalytic domains [1,39-42], thus the catalysis and substrate binding sites of the PheDH from *B. halodurans* can be identified by the crystal structure and active sites of the *Rs*PheDH from *Rhodococcus* sp. M4 (PDB: 1C1D and 1BW9) have been reported and confirmed [32, 33]. The amino acid sequence of PheDHs was compared (Fig. 1), and it showed that the enzymes have high similarity, indicating that the enzymes’ conservative regions were similar. Comparing with the previous reports [1, 32, 33, 35], the substrate binding and catalysis sites of the PheDH was confirmed. The amino acid residues surrounding the L-Phe substrate binding pocket, 49L-50G-51G, 74M,77K, 122G-123T-124D-125M, 275N, 305L,nd 89K of the PheDH, were identified (Fig. 1) and the locations of these sites were showed on the three-dimensional structure of the PheDH (Figs. 2A and 2B). The two sites 89K andhich here correspond to the site of the LeuDH [39], were essential to the catalytic mechanism of the PheDH.

### Transposition of PheDH Mutations

By using site-directed mutagenesis, an aspartic acid residue and a leucine residue were introduced into the 113 and 276 positions respectively, which was verified by sequencing. The superposition of mutation pET-28a-E113D-N276L was obtained using the pET-28a-E113D as the template with the same method. The enzyme activity and substrate spectrum of the purified E113D, N276L, and E113D-N276L was measured and compared. Furthermore, the pertinent kinetic parameters, substrate specificity and stability of PheDH mutants were further studied.

### Expression, Purification and Activity of the Enzymes

**Fig. 3:**
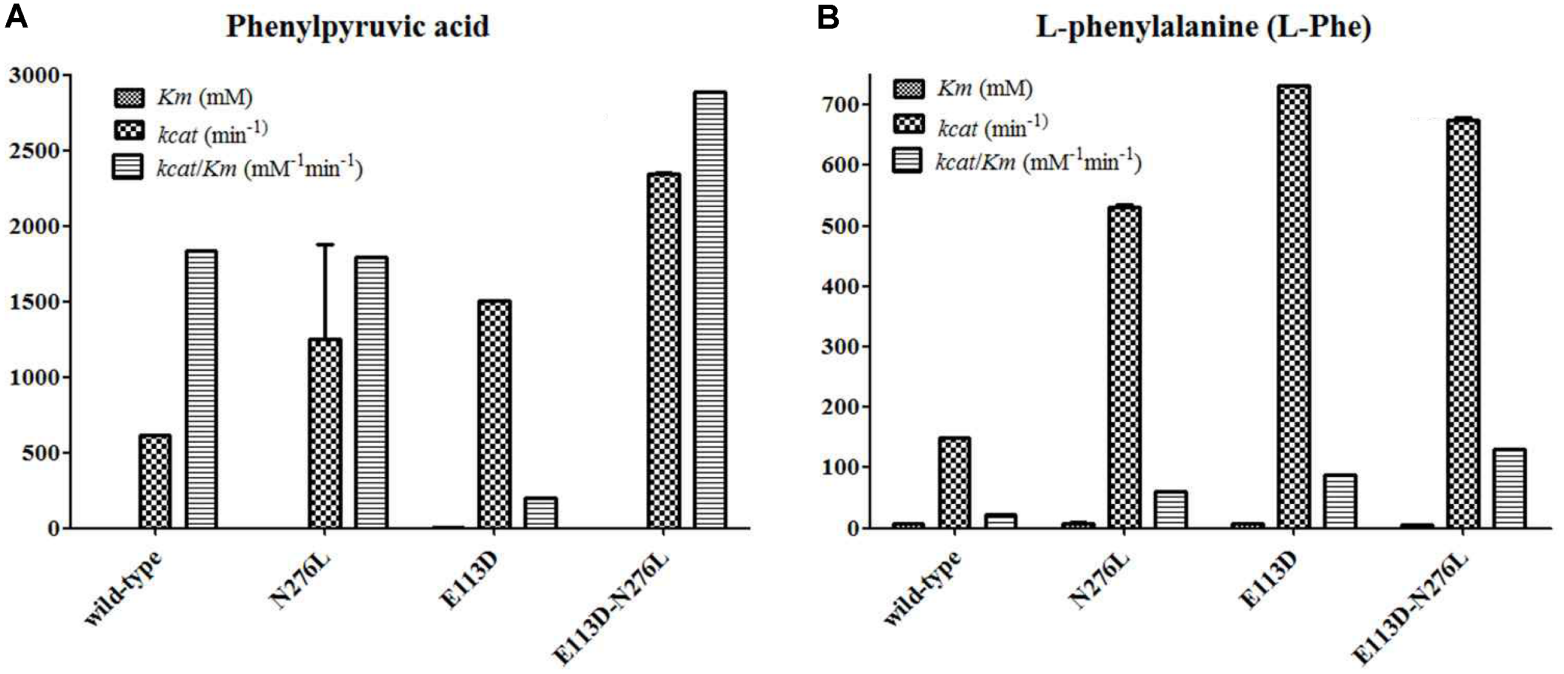
Steady-state kinetic parameters for the reductive amination of phenylpyruvate acid, and oxidative deamination of L-phenylalanine. (**A**) Steady-state kinetic parameters for the reductive amination of phenylpyruvate acid. (**B**) Steady-state kinetic parameters for the oxidative deamination of L-phenylalanine. The PDH and mutants had no activity with the D-Phe as substrate. Data represent the mean ± standard deviation of triplicate samples.

After purification, the PheDH and mutants were harvested and showed a single band in SDS-PAGE and all purified enzymes shared a similar molecular mass (Fig. S2). The steady-state kinetic parameters of the mutants and their parent were determined at 25°C and at a substrate-of-interest concentration ranging from 1.0-100 mM (Fig. 3 and Table S2). In the reductive amination section (phenylpyruvate acid as the substrate), the mutant E113D showed 2.44-fold increment in *kcat*, the better catalytic properties compared with the parent, while the mutant E113D-N276L showed 3.80-fold increase in *kcat* and approximately 1.58-fold increase in *k_cat_*/*K*m, indicating that the catalytic efficiency was considerably increased by replacing the glutamic acid with aspartic acid at the 113 position and substituting the asparagine with leucine at the 276 site. Moreover, the mutant E113D-N276L showed 3.80-fold increase in *kcat*, suggesting that the catalytic rate was considerably increased by replacing the glutamic acid with aspartic acid at the 113 position and substituting the asparagine with leucine at the 276 site. For oxidative deamination reaction (L-phenylalanine as substrate), the catalytic efficiency of all three mutations was significantly improved and the catalytic efficiency of the mutants E113D, N276L, and E113D-N276L was found to be 4.08, 2.81, and 6.06-fold higher than that of the wild type, respectively. The mutant E113D-N276L showed 6.13%decrease in *K*m and 4.53-fold increase in *kcat*, resulting in approximately 6.06-fold increase in (*kcat*/*Km*), indicating that the 113 site is crucial to enhance the enzyme activity. The decrease of the *Km* can also be attributed to the increase of enzyme and substrate affinity by the superimposed effect.

The *Km* for phenylpyruvic acid of the mutant enzymes K69A/K81A, K69A, and F124M/V125S/H126I/A127I/A128Y/R129Q from *Thermoactinomyces intermedius* [43, 44] was 8.62-fold, 10.22-fold, and 28.33-fold above that of the E113D-N276L, respectively, suggesting the higher affinity with the substrate of the E113D-N276L. The Km for L-Phe of the mutation G124A/E313G from *Lysinibacillus sphaericus* [22] was 1.87-fold above that of the E113D-N276L, suggesting that the E113D-N276L had higher affinity with substrate.

The catalytic efficiency value of the E113D-N276L was nearly 3.22-fold greater than the best mutation of 40.8 min^-1^ for the previous saturation mutagenesis of PheDH [22], indicating that the catalytic efficiency was significantly enhanced by replacing the glutamic acid with aspartic acid at the 113 position or substituting the asparagine with leucine at the 276 site alone or in combination. To our knowledge, this is the first study to report significantly improving the catalytic efficiency and creating a new PheDH from the PheDH of the marine microorganism *B. halodurans*. The increase of the catalytic efficiency can be attributed to the following factors: (i) the 113 site is too far away from the center of the enzyme structure, resulting in no mutation in the catalytic active site (Figs. 1 and 2, Figs. S4 and S5). This is confirmed by the N276L with low activity as it located at the center of the enzyme structure;(ii) the three-dimensional structure and docking results (Figs. 1, 2, S4, and S5) of PheDH show that the asparagine (276 site) probably locates at the end of a substrate channeling or an active channel; (iii) as the relative abundance of asparagine, leucine, glutamic acid and aspartic acid are 9.9, 7.8, 10.8, and 9.9 respectively, which may hinder the enzyme in combining with the substrate [36]. The greater the relative abundance of amino acids, the greater the steric hindrance, suggesting a higher likelihood of blocking the binding of the substrate to the enzyme [36].

### Stability of Temperature and pH Value

The E113D showed the best thermostability compared to the wide type and the other two mutants. After 100 min incubation under pH 7.5 (Fig. S3a), the E113D retained over 90% of its maximal activity from 35°C to 65°C and over 50%at 55°C , while the *Microbacterium* sp. PheDH lost half of the enzyme activity after an hour of incubation at 65°C [45]. Like the parent enzyme, E113D showed the best pH stability and it kept over 90% activity over a pH range from 7.0 to 9.5 for 48 h at 4°C (Fig. S3b). However, the N276L showed relatively weak pH but was stable at 9.0 and 9.5. For the reductive amination, the wide type and all three mutants showed the best thermostability and they maintained over 80% of their maximal activity from 35°C to 60°C (Fig. S3c), indicating superiority when compared with the *Microbacterium* sp. PheDH [45]. We also found that the mutant showed better pH stability than the wide type (Fig. S3d).

### Substrate Specificity

The substrate spectrum of the new PheDH was evaluated by determining the deamination and amination activities toward various amines and ketones, ranging from small aliphatic ketones such as 5-methyl-2, 3-hexanedione to larger aromatic ketones with additional functionality, such as phenoxy-2-propanone. The new PheDH showed enhanced activity toward cyclic ketones (Table 1).

These mutations showed activity for the analogous ketone as the amino acid residues at the mutant site were close to the substrate-binding pocket site 275N or directly interacted with some substrate-binding pocket sites 122G-123T-124D-125M [1, 32, 33, 35]. The mutants were further characterized for amination of the analogous ketone. The simplest ketone analog, phenylpyruvate, was selected as the model substrate replacing the natural substance of PheDH, phenylacetone, since it is regulated as a Schedule II controlled substance and is not readily available [46]. Unlike the AmDH which was obtained by Michael J. Abrahamson *et al*. [35], the most active ketones, phenoxy-2-propanone and 3-methylcyclohexanone, are not fully methyl ketones. The most active ketones for the three mutants were different and the best substrate for the E113D, N276L and E113D-N276L, were 2-methylcyclohexanone, 3-methylcyclohexanone and 5-methyl-2,3-hexanedione, respectively. For E113D-N276L, the specific activity is further detected in the large differences in activity toward cyclohexanone (33 mU/mg) versus 2-methylcyclohexanone and 3-methylcyclohexanone (0 mU/mg): despite their sharing similar structure, the methyl ketone 2-methylcyclohexanone and 3-methylcyclohexanone exhibited almost no activity.

The original scaffold, PheDH from *B. halodurans* for the mutants that accept the analogous ketone, shares a reasonable sequence identity to other amino acid dehydrogenases. It is also well known that amino acid dehydrogenases share prominent sequence similarities in the coenzyme binding and catalytic domains [39, 40]. The pET-28a-E113D has the same amino acid residue with the PheDH from *Oceanobacillus iheyensis* at the 113 position (Fig. 1). These residues, Glu113, directly interact with some substrate-binding pocket sites, 122G-123T-124D-125M, and Asn276, a close neighbor to the substrate-binding pocket sitehich interacts with the carboxy moiety of the wild-type ligand and is involved in binding the cofactor (Figs. 1 and 2), were selected as the point for mutation. Influential mutations identified in LeuDH-AmDH were evaluated to develop PheDH-AmDH activity [8, 35] while the role of a single site has not been explored. Analogical substitutions in the PheDH were identified to directly create a mutant that accepts the analogous ketone (Table 1). The variant E113D was the best mutant of the known mutations as it showed high activity on reductive amination of ketones, suggesting 113 amino acid residues play an important role in changing the enzyme substrate spectrum and creation of a mutant. All three mutants interact with the wild-type substrate at the carboxyl moiety, and their synergistic effects are reflected in the enormous increase (Fig. 3 and Tables 1, S2). The new PheDH still retains the natural PheDH activity, which is helpful for the investigation of the mechanism of the evolutionary progress from amino acid dehydrogenase to other dehydrogenases.

In conclusion, we have resoundingly developed three mutants by starting with a PheDH as template and subsequent active-site-targeted, site-specific mutagenesis. The PheDH active site with bound L-Phe and surrounding residues was confirmed. It’s worth noting that the catalytic efficiency of the ideal mutant E113D-N276L was found to be approximately 6.06 and 1.58-fold higher than that of the wild type in the oxidative deamination and reductive amination reaction, respectively. The mutants exhibited high activity toward a number of benzylic and aliphatic ketone substrates. With the advantages of high activity and selectivity as well as excellent thermal stability and broad spectrum, the developed multifunctional enzymes provide efficient biocatalysts that can be utilized for the synthesis of chiral compounds.

## Supplemental Materials



Supplementary data for this paper are available on-line only at http://jmb.or.kr.

## Figures and Tables

**Table 1:** Substrate profiles of top amination and deamination mutants.

Substrate	Activity (U/mg = μmol/min·mg)		

	N276L	E113D	E113D-N276L
Phenylpyruvic acid^a^	24.33±0.10	22.87±0.21	38.00±0.06
Phenoxy-2-propanone^a^	1.22±0.03	n.m.^c^	n.m.^c^
3-Methylcyclohexanone^a^	1.67±0.03	0.41±0.04	n.m.^c^
2-Methylcyclohexanone^a^	0.76±0.04	0.69±0.10	n.m.^c^
Cyclohexanone^a^	1.06±0.01	0.69±0.03	0.33±0.01
5-Methyl-2,3-hexanedione^a^	0.46±0.01	0.55±0.05	1.31±0.03
L-Phenylalanine^b^	9.58±0.81	11.57±1.00	24.24±0.39
2-Methyl-benzylamine^b^	n.m.^c^	n.m.^c^	0.66±0.05
1,4-Dimethylpentylamine^b^	n.m.^c^	0.14±0.09	n.m.^c^

^a^Amination in 500 mM NH_3_·H_2_O/NH_4_Cl, 100 mM glycine-KCl-KOH buffer, 100 microM NADH, 20 mM of the ketone substrate with 0.01 mg enzyme 25°C.

^b^Deamination in 50 mM glycine-KCl-KOH buffer (pH 10.4), with 2 mM NAD^+^ with 20 mM of the amine substrate of interest with 0.01 mg enzyme 25°C.

^c^n.m.= not measurable, <0.1 mU mg^-1^ .
