# Supplementary material for: Improving Catalytic Efficiency and Changing Substrate Spectrum for Asymmetric Biocatalytic Reductive Amination
Source: J Microbiol Biotechnol. 2019 Sep 18;30(1):146–54. doi: 10.4014/jmb.1907.07015 (PMC9728165; doi:10.4014/jmb.1907.07015)
Supplement: Supplementary file 1 [file JMB-30-1-146-supple.pdf]

J. Microbiol. Biotechnol.
https://doi.org/10.4014/jmb.1907.07015

jmb

Support information

Figures

Fig.S1. Multiple sequence comparison of the PheDH and LeuDH by ClustalW. The amino acid sequence of the leucine dehydrogenase from *Bacillus stearothermophilus* was obtained by NCBI database (http://www.ncbi.nlm.nih.gov/guide/) and pre-reported (Sekimoto et al. 1994).

Fig.S2. 12% SDS-PAGE analysis of the purified proteins. Lane 1: purified E113D with His-tag. Lane 2: purified N276L with His-tag. Lane 3: purified E113D-N276L with His-tag. Lane 4: protein marker. Lane 5: purified PheDH with His-tag. The protein molecular weight ladder is Unstained Protein Molecular Weight Marker (Fermentas, Canada).

Fig.S3. Effects of temperature and pH on enzyme stability. (a) Effect of temperature on the stability of the PheDH and mutants of reductive amination with PheDH (Black circles, ●), N276L (black squares, ■), E113D (prismatic, ◆) and E113D-N276L (del operator, ▼). At the optimal pH 7.0, the purified enzyme was pre-treated at a different temperature for 100 min. The activity of the enzyme without pre-incubation was defined as 100%. (b) Effect of pH on the stability of the PheDH and mutants of reductive amination with PheDH (Black circles, ●), N276L (black squares, ■), E113D (prismatic, ◆) and E113D-N276L (del operator, ▼). The pH stability of the PheDH and mutants was determined by incubating the enzymes at a different pH at 4 ℃ for 48 h. Then assays were conducted in the standard conditions and the enzyme activity without pre-treatment was taken as 100%. (c) Effect of

temperature on the stability of the PheDH and mutants of oxidative deamination with PheDH (Black circles, ●), N276L (black squares, ■), E113D (prismatic, ◆) and E113D-N276L (del operator, ▼). At the optimal pH 7.0, the purified enzyme was pre-treated at a different temperature for 100 min. The activity of the enzyme without pre-incubation was defined as 100%. (d) Effect of pH on the stability of the PheDH and mutants of oxidative deamination with PheDH (Black circles, ●), N276L (black squares, ■), E113D (prismatic, ◆) and E113D-N276L (del operator, ▼). The pH stability of the PheDH and mutants was determined by incubating the enzymes at a different pH at 4 ℃ for 48 h. Then assays were conducted in the standard conditions and the enzyme activity without pre-treatment was taken as 100%. Error bars represent the standard deviation.

Fig.S4. The location of the mutation sites on the PheDH three-dimensional structure. The sites of 113 and 276 were indicated on the three-dimensional structure, respectively.

Fig.S5. Modeled three-dimensional structure of the PheDH and mutants. (a) the three-dimensional structure of the wild type enzyme; (b) the three-dimensional structure of the E113D; (c) the three-dimensional structure of the N276L; (d) the three-dimensional structure of the E113D-N276L. The sites of site-directed mutagenesis were indicated on the three-dimensional structure. The three-dimensional structure was generated using the EasyModeller4.0.

Table.S1.Primers used for plasmid construction and the site-directed mutagenesis.

Table.S2. Steady-state kinetic parameters for the reductive amination of phenylpyruvate

acid, and oxidative deamination of L-phenylalanine.

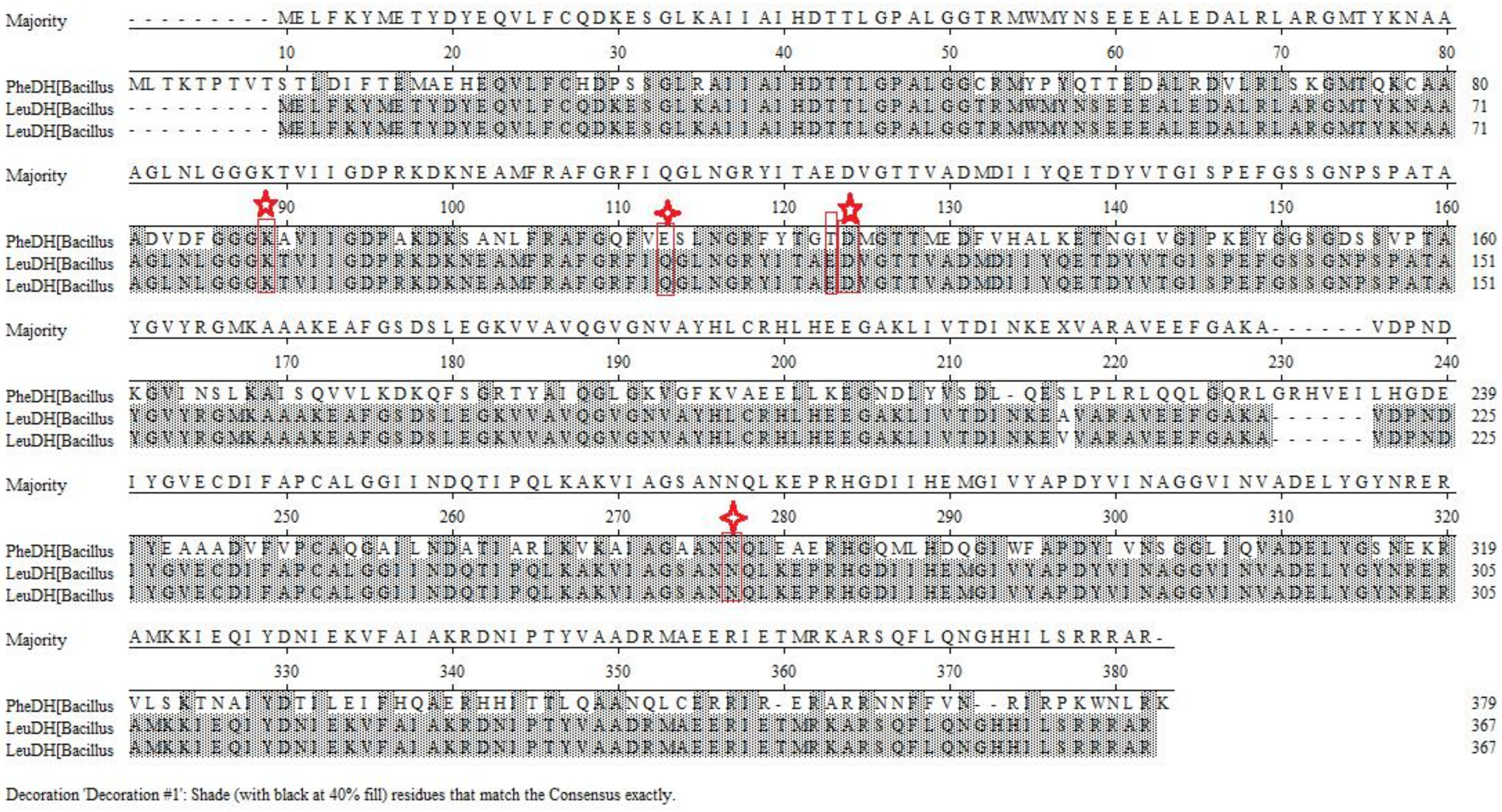

Figure S1 Multiple sequence comparison of the PheDH and LeuDH by ClustalW

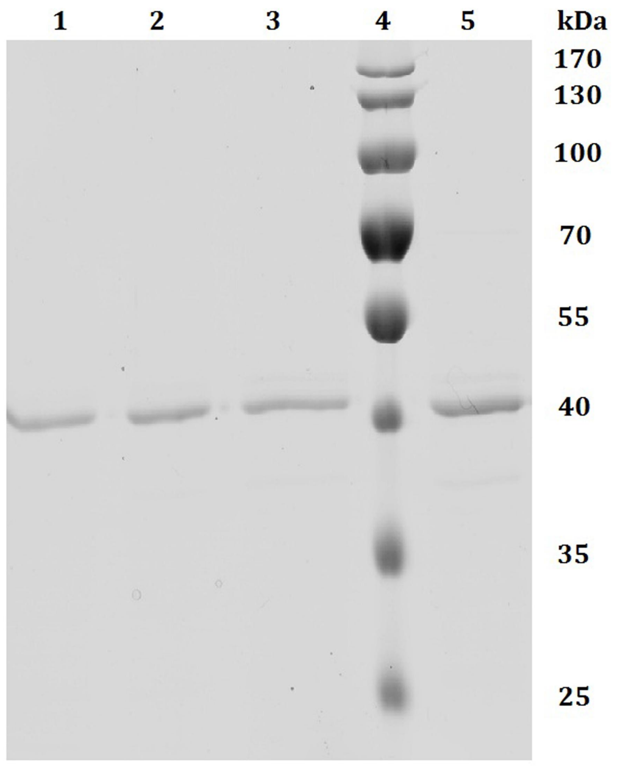

Figure S2 12% SDS-PAGE analysis of the purified proteins

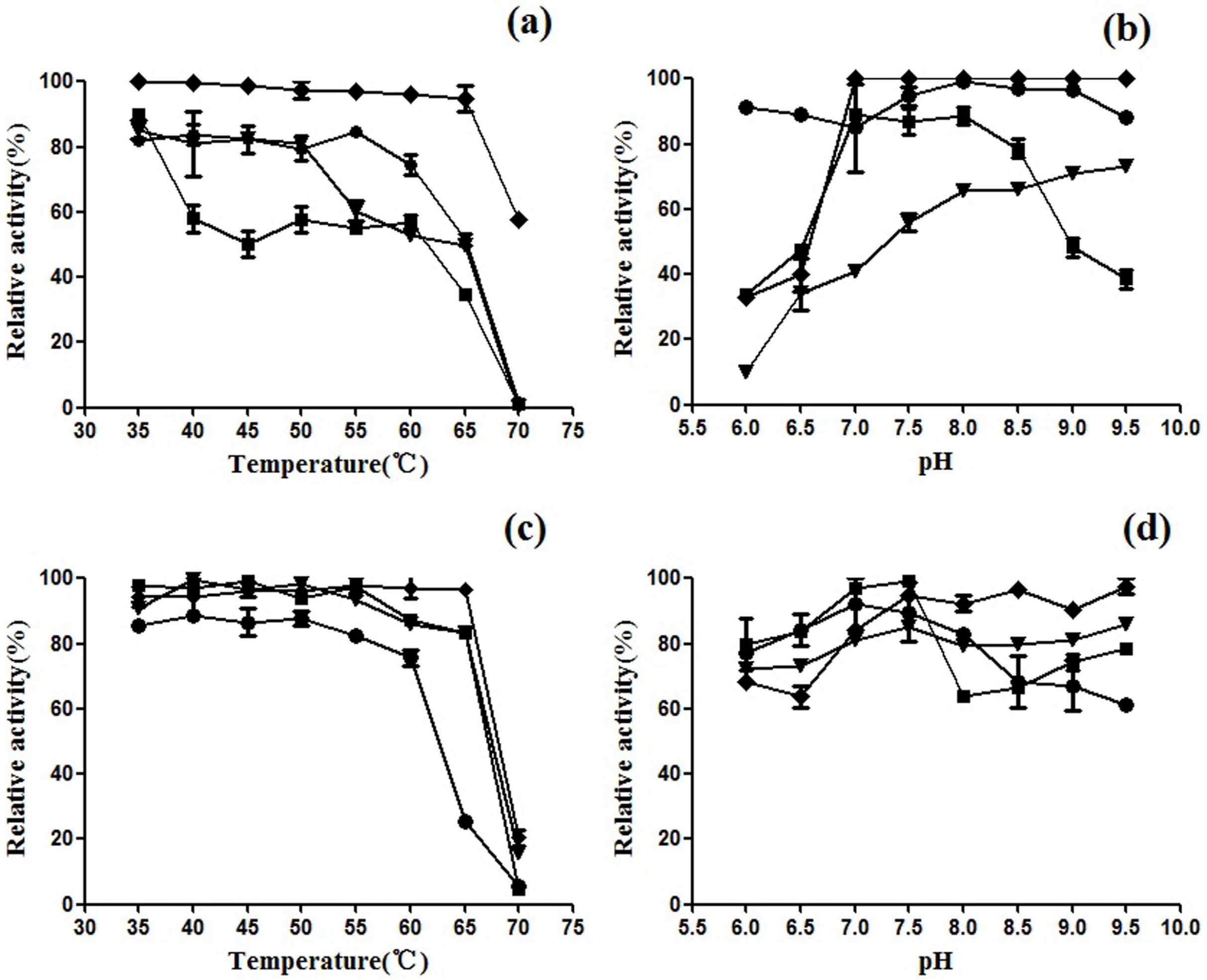

Figure S3 Effects of temperature and pH on enzyme stability

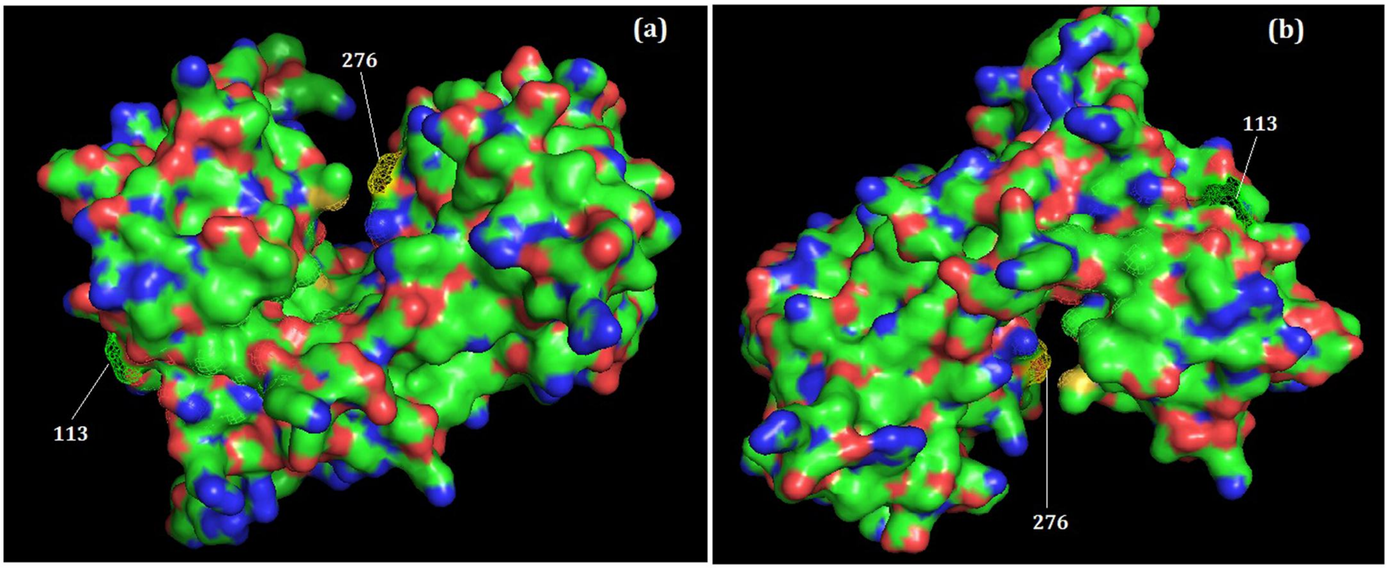

Figure S4 The location of the mutation sites on the PheDH three-dimensional structure

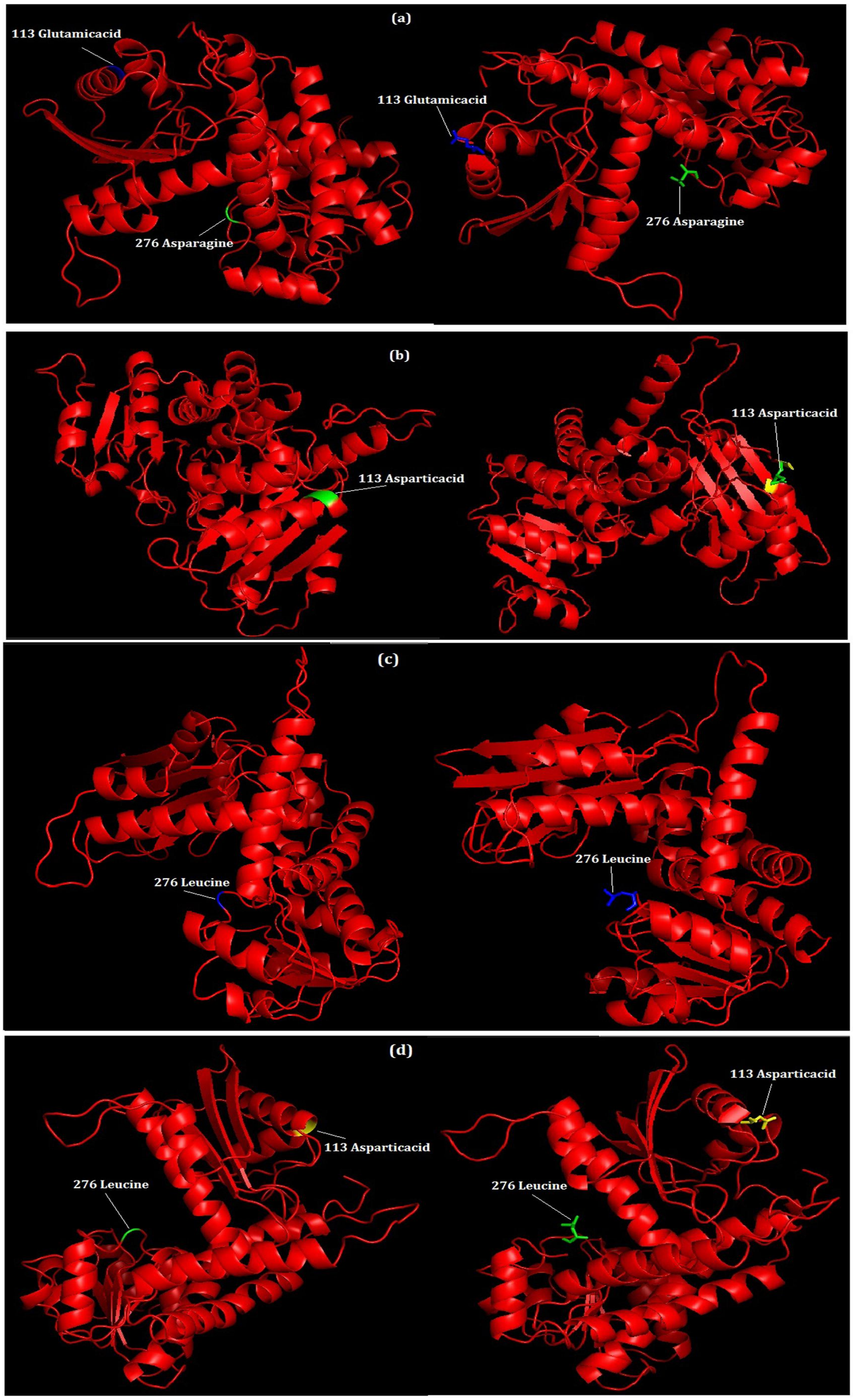

Figure S5 Modeled three-dimensional structure of the PheDH and mutants

Tables

Table S1 Primers used for plasmid construction and the site-directed mutagenesis

Primers	5′ to 3′
E113D-F	GGACAATTTGTTGATTCTTTGAATG
E113D-R	ATCAACAAATTGTCCAAACGCCCTA
N276L-F	GGAGCGGCAAACCTTCAGCTAGAAG
N276L-R	AAGGTTTGCCGCTCCGGCAATAGCC

Note: The mutated site is underlined.

Table S2 Steady-state kinetic parameters for the reductive amination of phenylpyruvate acid, and oxidative deamination of L-phenylalanine

Enzyme	Substrate	K_m (mM)	k_{cat} (min^{-1})	k_{cat}/K_m ($mM^{-1}min^{-1}$)
wild-type	Phenylpyruvic acid	0.337±0.05	617.78±1.0	1833.71
N276L		1.05±0.1	1879.26±2.3	1794.90
E113D		7.607±0.2	1507.42±1.0	198.16
E113D-N276L		0.812±0.01	2345.11±4.0	2889.14
wild-type	L-phenylalanine (L-Phe)	6.88±0.41	149.08±0.21	21.68
N276L		8.71±0.2	530.61±4.12	60.93
E113D		8.28±0.4	731.58±0.3	88.41
E113D-N276L		5.14±0.3	675.58±3.3	131.36

Note: The PDH and mutants had no activity with the D-Phe as Substrate. Data represent the mean ± standard deviation of triplicate samples.
